# Supplementary material for: Coordinated human-exoskeleton locomotion emerges from regulating virtual energy
Source: PLoS One. 2025 Jan 22;20(1):e0292334. doi: 10.1371/journal.pone.0292334 (PMC11753675; doi:10.1371/journal.pone.0292334)
Supplement: S1 Appendix — (PDF) [file pone.0292334.s001.pdf]

Coordinated Human-Exoskeleton Locomotion Emerges from Regulating Virtual Energy

Rezvan Nasiri[1], Hannah Dinovitzer[1], Nirosh Manohara[1], and Arash Arami[1,2*]

1 Department of Mechanical and Mechatronics Engineering, University of Waterloo, Waterloo ON, Canada
2 Toronto Rehabilitation Institute (KITE), University Health Network, Toronto, ON, Canada.

* arash.arami@uwaterloo.ca

Supporting information

The appendix includes the experimental results in terms of muscle activities, joint kinematics, limit cycles, toe clearance, ground reaction forces, control objectives, questionnaire, and its results. In addition, it contains experimental setup figures, feasible limit cycle examples, and virtual energy regulator gait phase estimation results. The presented results in this appendix are mainly for $0.6m/s$ and $0.75m/s$ and the results for $0.85m/s$ are reported in the main body of the paper.

Ground reaction forces

Figure G compare the 3D GRF in the Natural, Passive, and Active conditions at $0.85m/s$ for a representative participant. Also, this figure compare the Passive and Active conditions w.r.t. the Natural walking in terms of correlation coefficient and RMS of deviations from Natural at $0.85m/s$ across all participants. We observed similar patterns as in **Figure G** at other speeds; see **Figure E** and **Figure F**.

In Passive, GRF in z direction (vertical component) has an early stance peak which is similar to crouch walking; this is due to the added weight of the exoskeleton. VER successfully corrects this semi-pathologic behavior in z-direction, however, it cannot effectively change the GRF profiles in x-direction; i.e., mediolateral direction, as our exoskeleton has no actuation effecting the mediolateral motion. Nevertheless, the GRF in the sagittal plane, z- and y-directions, are properly modified by VER and matches the GRF in Natural condition. It must be noted that as the Indego exoskeleton has only one degree of freedom at hip and knee joint and no actuation at ankle joint, our implemented VER has no control over the ankle joint and cannot further improve the vertical GRF.

Questionnaire

The participants were asked to fill a questioner after each trail by scoring their comfort, safety, stability, effort, and fatigue time for Passive and Active conditions. The questions are as follows:

1 Please score your **comfort** from 0 to 10: 10 indicates your comfort in normal walking and 0 indicates minimum possible comfort.

2 Please score your **safety** from 0 to 10: 10 indicates your safety in normal walking and 0 indicates minimum possible safety.

3 Please score your **stability** from 0 to 10: 10 indicates your stability in normal walking and 0 indicates minimum possible stability.

4 Please score you **effort** from 0 to 10; 0 indicates that there is almost no effort, 10 indicates the most possible effort.

5 Please score your **fatigue**: how long more can you walk on the treadmill? Any number between 0 to 20 min or more than 20 min.

The questioner results for walking at $0.6m/s$ and $0.85m/s$ are presented in the main body, and the results at $0.75m/s$ are presented in **Figure J**.

VER as a gait phase estimator

Based on **Def. 1** in section "VER mathematical foundation" of the main body, VER phase (θ) is monotonically decreasing during the gait. Particularly, in the hip joint for both desired and natural limit cycles, this property is always satisfied. Thus, the representation of hip limit cycle phase (θ) in VER is unique through the gait cycle. Accordingly, the gait phase (G) can be estimated using the VER hip limit cycle phase (θ_h) as $G = \Gamma(\theta_h)$ where Γ is a monotonically increasing and bijective function of the phase (θ). We introduced this property of VER in [39] for the first time, and here, it is extensively experimented at all three speeds; see **Figure L**.

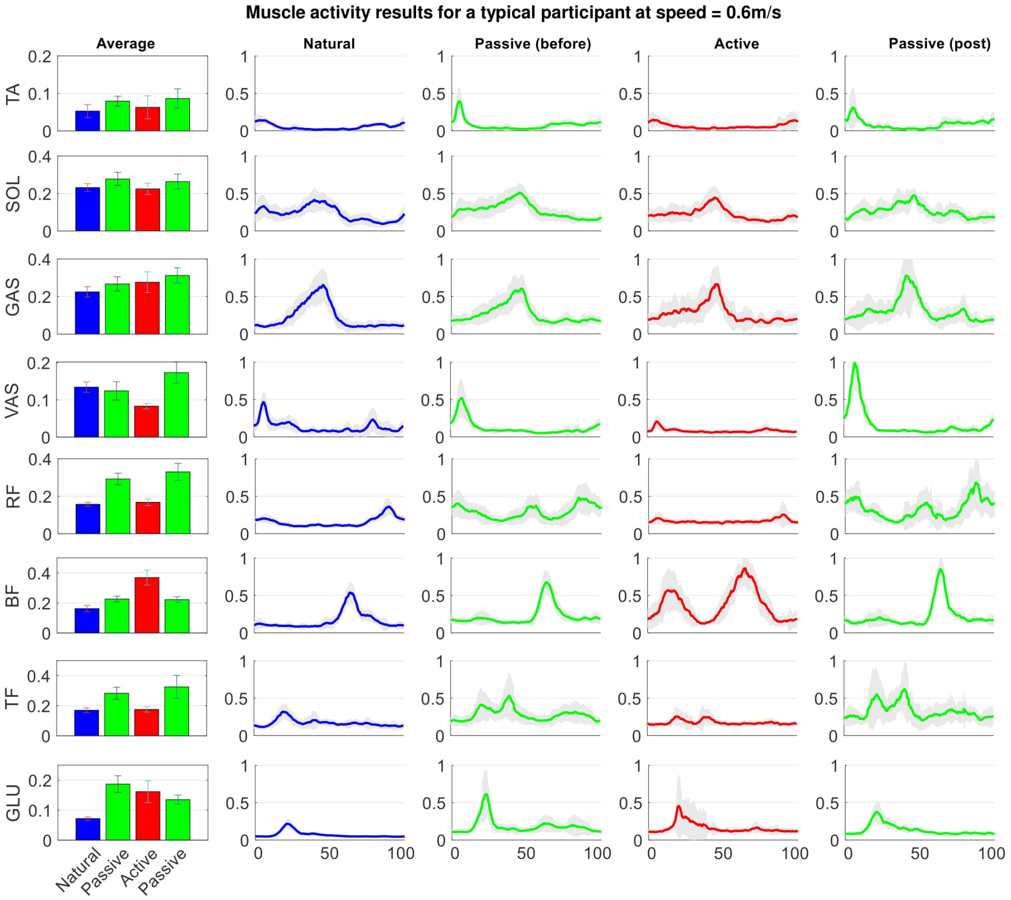

Figure A. Muscle activation pattern comparison at $0.6m/s$**.** Comparison between muscle activation patterns and average muscle activity for a representative participant in four different conditions; Natural, Passive before, Active, and Passive post. The selected muscles for data collection are: Tibialis Anterior (TA), Soleus (SOL), Gastrocnemius Medialis (GAS), Vastus Medialis (VAS), Rectus Femoris (RF), Tensor Fascia (TF), Biceps Femoris (BF), and Gluteus Maximus (GLU). For this typical participant, VER (Active condition) results in average muscle effort reduction of 14.1% compared to Passive condition (when the controller is off). For all participants, VER leads to $14.4\% \pm 4.0\%$ average muscle effort reduction ($p = 0.0156$, *two-sided Wilcoxon signed rank test*).

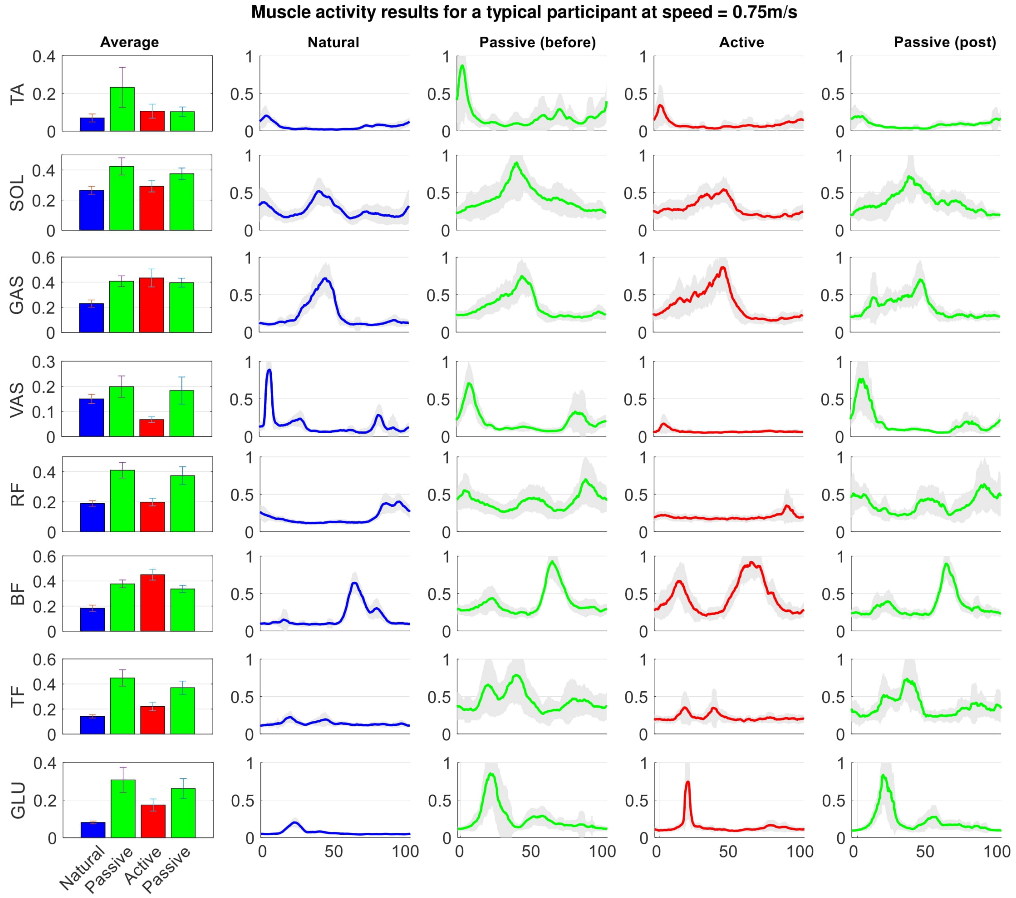

Figure B. Muscle activation pattern comparison at $0.75m/s$**.** Comparison between muscle activation patterns and average muscle activity for a representative participant in four different conditions; Natural, Passive before, Active, and Passive post. The selected muscles for data collection are: Tibialis Anterior (TA), Soleus (SOL), Gastrocnemius Medialis (GAS), Vastus Medialis (VAS), Rectus Femoris (RF), Tensor Fascia (TF), Biceps Femoris (BF), and Gluteus Maximus (GLU). For this typical participant, VER (Active condition) results in average muscle effort reduction of 28.6% compared to Passive condition (when the controller is off). For all participants, VER leads to 17.7% $\pm$ 6.2% average muscle effort reduction ($p = 0.0156$, *two-sided Wilcoxon signed rank test*)

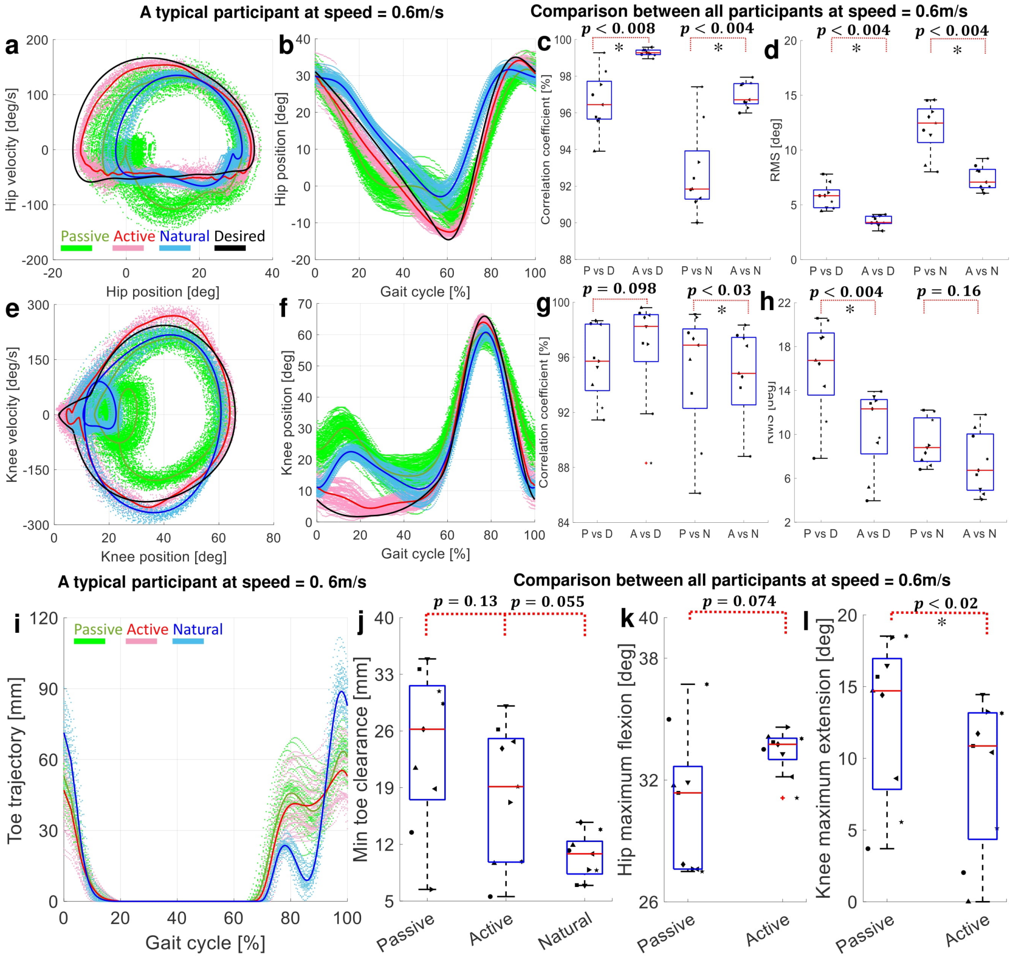

Figure C. Kinematic comparison at $0.6m/s$ (a,b,e,f) compare the Desired trajectory and limit cycle with Passive, Active, and Natural conditions at hip and knee joints for a representative participant. (c,d,g,h) compare the correlation coefficient and RMS of deviations from the Desired and Natural trajectories with Active and Passive conditions at hip and knee joints across all participants. (i) shows the toe clearance trajectory for the typical participant, and (j) compares minimum toe clearance of Passive, Active, and Natural conditions across all participants. (k,l) compare Passive and Active conditions in terms of hip(knee) maximum swing(stance) flexion(extension) across all participants; zero angle corresponds to a fully extended knee.

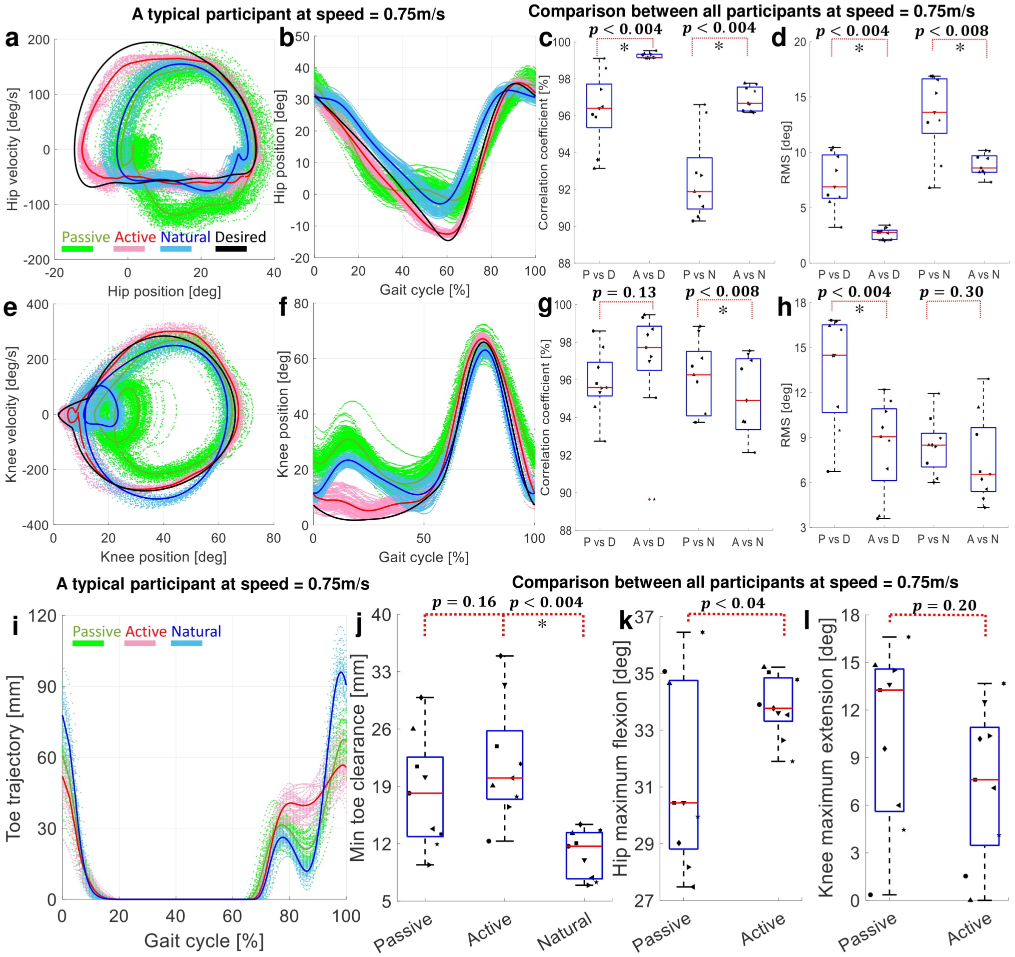

Figure D. Kinematic comparison at $0.75 m/s$ (a,b,e,f) compare the Desired trajectory and limit cycle with Passive, Active, and Natural conditions at hip and knee joints for a representative participant. (c,d,g,h) compare the correlation coefficient and RMS of deviations from the Desired and Natural trajectories with Active and Passive conditions at hip and knee joints across all participants. (i) shows the toe clearance trajectory for the typical participant, and (j) compares minimum toe clearance of Passive, Active, and Natural conditions across all participants. (k,l) compare Passive and Active conditions in terms of hip(knee) maximum swing(stance) flexion(extension) across all participants; zero angle corresponds to a fully extended knee.

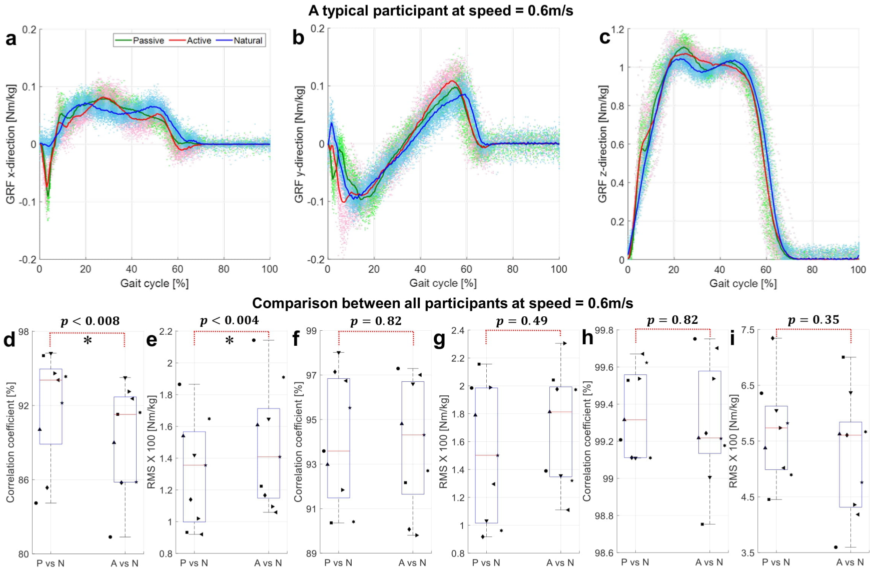

Figure E. GRF comparison at $0.6m/s$ (a-c) compare Natural 3D GRF profiles with GRF profiles in Passive and Active conditions for a representative participant. (d-i) compare the similarity of Natural 3D GRF profiles with Active and Passive conditions across all participants. The similarity is measured in terms of correlation coefficient and RMS of deviations from Natural GRD profiles.

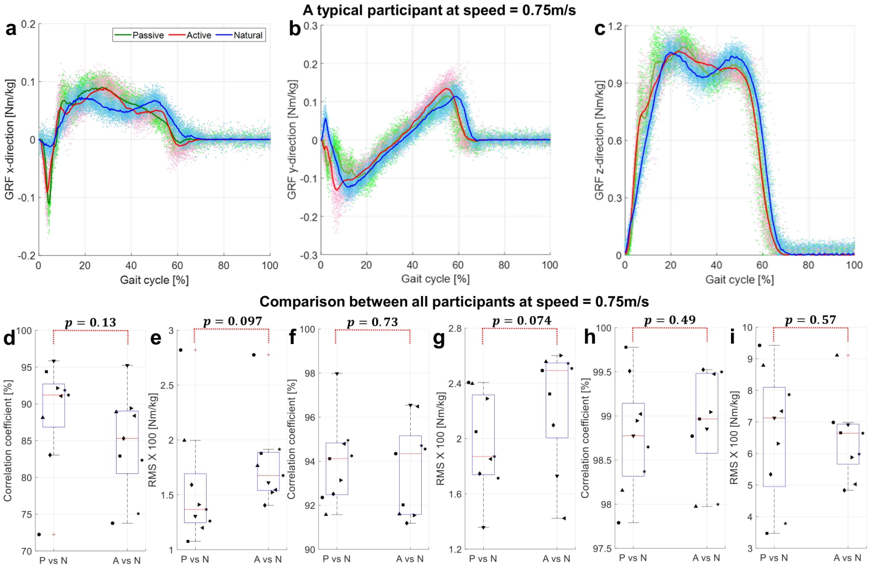

Figure F. GRF comparison at $0.75m/s$ (a-c) compare Natural 3D GRF profiles with GRF profiles in Passive and Active conditions for a representative participant. (d-i) compare the similarity of Natural 3D GRF profiles with Active and Passive conditions across all participants. The similarity is measured in terms of correlation coefficient and RMS of deviations from Natural GRD profiles.

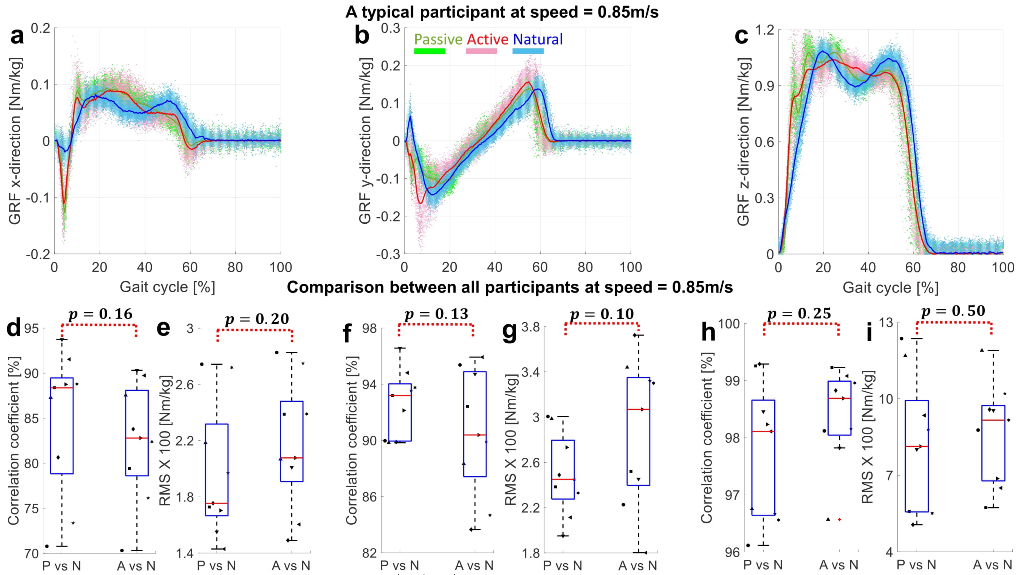

Figure G. GRF comparison at $0.85m/s$ (a-c) compare Natural 3D GRF profiles with GRF profiles in Passive and Active conditions for a representative participant. (d-i) compare the similarity of Natural 3D GRF profiles with Active and Passive conditions across all participants. The similarity is measured in terms of correlation coefficient and RMS of deviations from Natural GRD profiles.

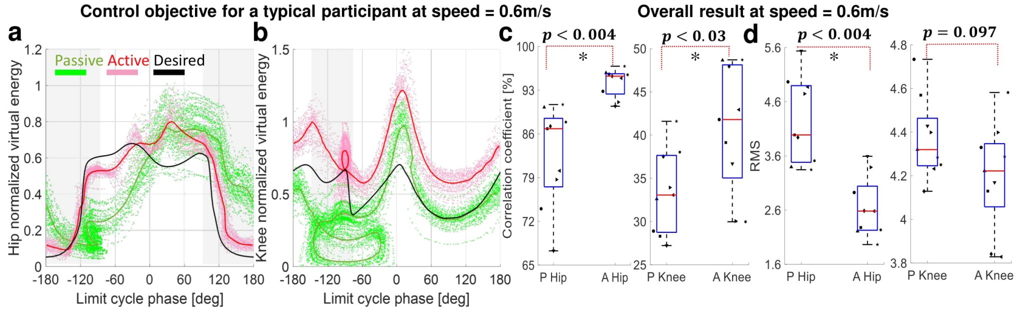

Figure H. VER control performance at $0.6m/s$. (a,b) describe virtual energy against the limit cycle phase where the gray backgrounds indicate the stance phase. (a,b) compare the desired virtual energy with virtual energy in two different conditions (Passive and Active) in hip and knee joints for a representative participant. (c,d) compare the similarity of the desired virtual energy with virtual energy in two different conditions (Passive and Active) for hip and knee joints across all participants. Similarity is measured in terms of correlation coefficient and RMS of deviation from the desired virtual energy.

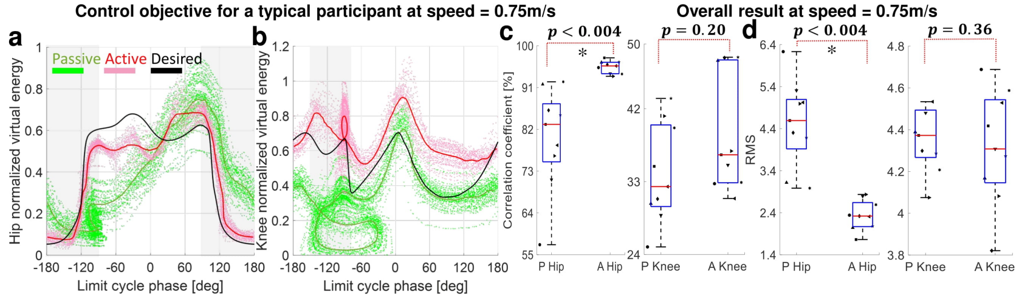

Figure I. VER control performance at $0.75m/s$. (a,b) describe virtual energy against the limit cycle phase where the gray backgrounds indicate the stance phase. (a,b) compare the desired virtual energy with virtual energy in two different conditions (Passive and Active) in hip and knee joints for a representative participant. (c,d) compare the similarity of the desired virtual energy with virtual energy in two different conditions (Passive and Active) for hip and knee joints across all participants. Similarity is measured in terms of correlation coefficient and RMS of deviation from the desired virtual energy.

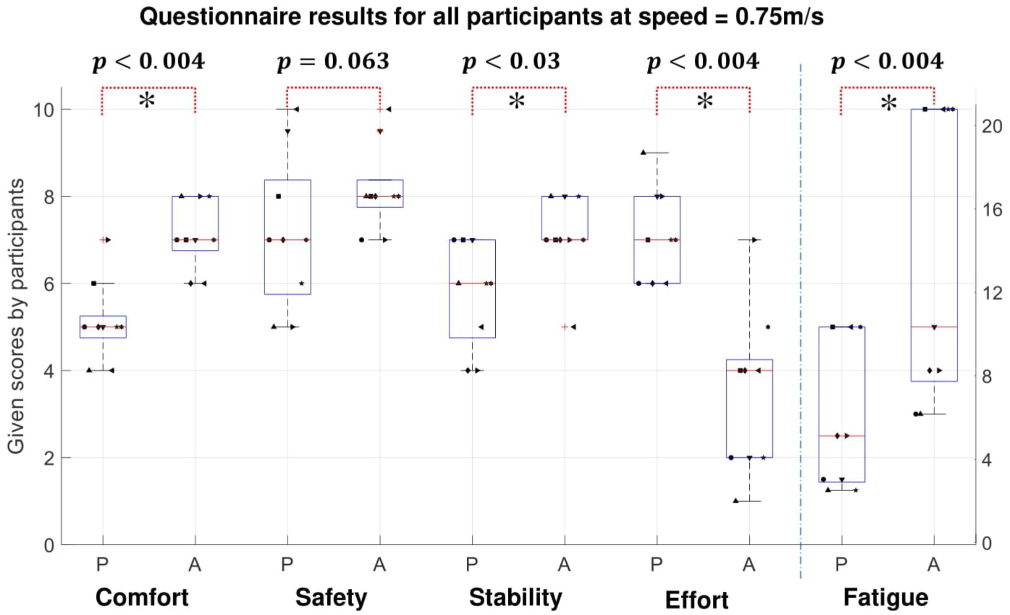

Figure J. The questionnaire results for all participants at $0.75 m/s$. The box plots compare Passive with Active condition in terms of comfort, safety, stability, effort, and fatigue time. The vertical axes for fatigue time is in right side of the plots. Participants perceived a significant improvement in most of indices. Perceived safety did not show any significant difference between the Passive and Active conditions.

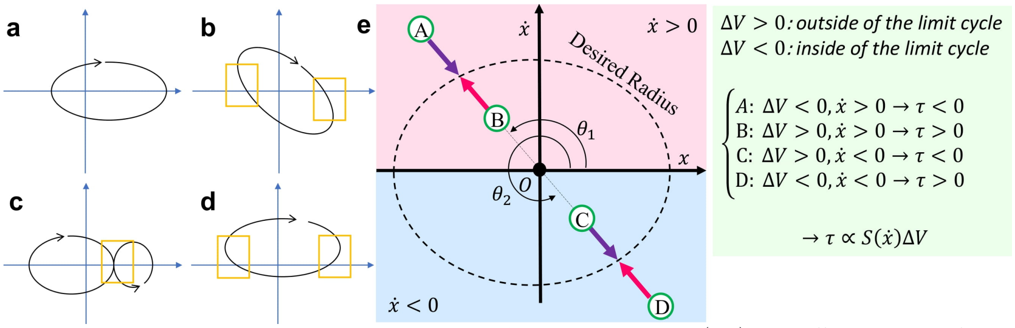

Figure K. Feasible limit cycles and illustration of how VER works. (a-d) The different shapes of limit cycles in all the plots where the vertical axis is velocity and the horizontal axis is position. With the exception of (a), all of the shapes are infeasible limit cycles. The orange boxes indicate the area of the limit cycles that violate feasibility conditions. (e) explains the gradient of VER control command w.r.t. the difference between virtual energy and the desired energy ($\Delta V = V_d - V(x, \dot{x})$ where the virtual energy is the square of radius from the considered center as $V(x, \dot{x}) = r^2$) and joint velocity ($\dot{x}$). As a result the VER control command is proportional to multiplication of both of velocity and difference in virtual energy $\tau \propto \Delta V S(\dot{x})$ where $S(\dot{x})$ has the same sign as $\dot{x}$; i.e., $\tau = P \Delta V S(\dot{x})$. Accordingly, the power applied by VER (P_a) by definition is its torque multiplied by the joint velocity as $P_a = \dot{x} S(\dot{x}) P \Delta V$ where we have $\dot{x} S(\dot{x}) > 0$. Hence, the applied power by VER is negative(positive) when the joint state is outside(inside) of the desired limit cycle. The magnitude of applied power is defined by the distance from the desired energy level. In other words, VER injects(dissipates) energy whenever the joint virtual energy ($V(x, \dot{x})$) is lower(higher) than the desired level defined over the desired limit cycle. The desired limit cycle on the other hand is the desired virtual energy level where VER tries to regulate the virtual energy on this boundary.

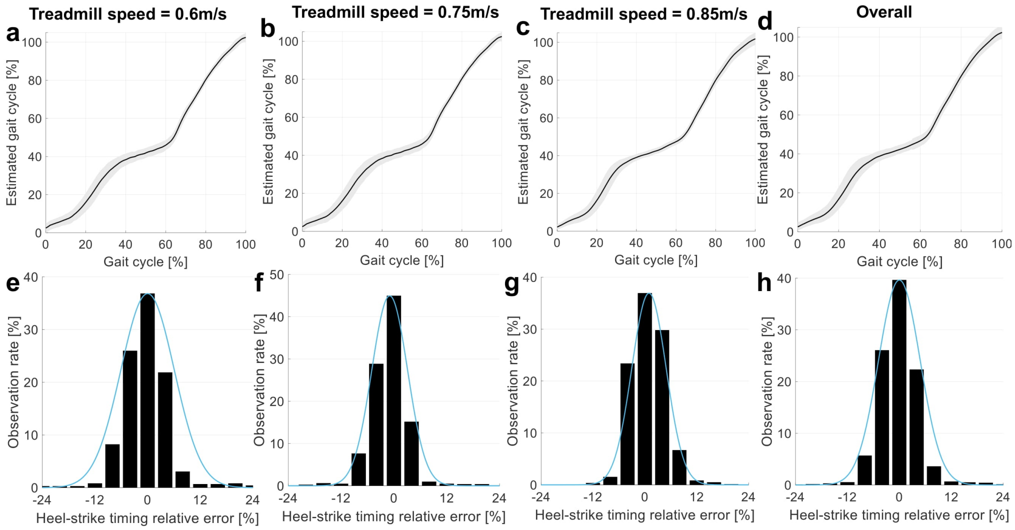

Figure L. VER as a gait phase estimator. (a-d) show the VER based estimated gait phase versus gait cycle percentage estimates obtained using the ground reaction force, starting from heel strike at three different speeds for all participants. The VER estimated gait phase is monotonically increasing with small standard deviations and remains identical across different speeds which means that it is a proper parameter to estimate the gait phase. (e-h) illustrate the observation rate of relative error for heel-strike event detection using the VER gait phase estimator at different speeds. Interestingly, the VER estimated gait phase is also an accurate measure for heel-strike event detection. All four distributions significantly pass One-sample Kolmogorov-Smirnov normal distribution test ($P^* \ll 0.00001$); error$\sim N_1(-0.1\%, 6.1\%)$, $N_2(-1.0\%, 4.1\%)$, $N_3(0.9\%, 4.0\%)$, and $N(-0.1\%, 4.8\%)$.
