# Supplementary material for: Coordinated human-exoskeleton locomotion emerges from regulating virtual energy
Source: PLoS One. 2025 Jan 22;20(1):e0292334. doi: 10.1371/journal.pone.0292334 (PMC11753675; doi:10.1371/journal.pone.0292334)
Supplement: Attachment — Submitted filename: Rebuttal Letter_final.pdf [file pone.0292334.s005.pdf]

The authors would like to thank the Associate Editor and Reviewers for their valuable comments on our manuscript, Submission ID PONE-D-23-27913, entitled "Coordinated human-exoskeleton locomotion emerges from regulating virtual energy". In the following, you may find our responses to the questions and comments of the reviewers. We addressed all the reviewers' comments one by one in this response letter and revised the manuscript accordingly. Changes are highlighted in the attached copy of the revised manuscript at the end of this document to facilitate finding the relevant modifications to each comment. The highlighted captions also show the revised captions and/or figures.

Best Regards,

Rezvan Nasiri, Hannah Dinovitzer, Nirosh Manohara, and Arash Arami

University of Waterloo

Journal Requirements

1. When submitting your revision, we need you to address these additional requirements. Please ensure that your manuscript meets PLOS ONE's style requirements, including those for file naming. The PLOS ONE style templates can be found at https://journals.plos.org/plosone/s/file?id=wjVg/PLOSOne_formatting_sample_main_body.pdf and https://journals.plos.org/plosone/s/file?id=ba62/PLOSOne_formatting_sample_title_authors_affiliations.pdf

We have used the PLOS ONE latex template for writing the paper and do our best to consider all PLOS ONE guidelines.

2. Please note that PLOS ONE has specific guidelines on code sharing for submissions in which author-generated code underpins the findings in the manuscript. In these cases, all author-generated code must be made available without restrictions upon publication of the work. Please review our guidelines at https://journals.plos.org/plosone/s/materials-and-software-sharing#loc-sharing-code and ensure that your code is shared in a way that follows best practice and facilitates reproducibility and reuse.

Our publication does not have any code accompanied.

3. Thank you for stating the following in the Acknowledgments Section of your manuscript:
This work is supported by NSERC Discovery, John R. Evans Leaders Fund (JELF)-Canada Foundation for Innovation, Ontario Research Fund (ORF), and the New Frontiers in Research Fund under Grant NFRFE-2018-01698.
We note that you have provided funding information that is not currently declared in your Funding Statement. However, funding information should not appear in the Acknowledgments section or other areas of your manuscript. We will only publish funding information present in the Funding Statement section of the online submission form.

Please remove any funding-related text from the manuscript and let us know how you would like to update your Funding Statement. Currently, your Funding Statement reads as follows:
-AA received NSERC Discovery under Grants RGPIN-2018-04850
-AA received New Frontiers in Research Fund - Exploration under Grants NFRFE2018-01698 and NFRFE2022-620
-AA received John R. Evans Leaders Fund Canadian Foundation for Innovation
-AA received Ontario Research Fund (ORF)
-HD received scholarship NSERC CGS-M.
The funders had no role in study design, data collection and analysis, decision to publish, or preparation of the manuscript.
Please include your amended statements within your cover letter; we will change the online submission form on your behalf.

We confirm that the provided format for data description is acceptable. We note that you have stated that you will provide repository information for your data at acceptance. Should your manuscript be accepted for publication, we will hold it until you provide the relevant accession numbers or DOIs necessary to access your data. If you wish to make changes to your Data Availability statement, please describe these changes in your cover letter and we will update your Data Availability statement to reflect the information you provide.

The authors will provide repository information after the paper acceptance.

4. PLOS requires an ORCID iD for the corresponding author in Editorial Manager on papers submitted after December 6th, 2016. Please ensure that you have an ORCID iD and that it is validated in Editorial Manager. To do this, go to 'Update my Information' (in the upper left-hand corner of the main menu), and click on the Fetch/Validate link next to the ORCID field. This will take you to the ORCID site and allow you to create a new iD or authenticate a pre-existing iD in Editorial Manager. Please see the following video for instructions on linking an ORCID iD to your Editorial Manager account: https://www.youtube.com/watch?v=_xcclfuvtxQ

Corresponding author's ORCID: 0000-0001-7609-6553 was added.

5. Please include captions for your Supporting Information files at the end of your manuscript, and update any in-text citations to match accordingly. Please see our Supporting Information guidelines for more information: http://journals.plos.org/plosone/s/supporting-information

The supporting document materials (which are mostly some figures) are already cited in the main body of the paper.

The captions and figure names are modified in the Supporting Materials.

6. Please review your reference list to ensure that it is complete and correct. If you have cited papers that have been retracted, please include the rationale for doing so in the manuscript text, or remove these references and replace them with relevant current references. Any

changes to the reference list should be mentioned in the rebuttal letter that accompanies your revised manuscript. If you need to cite a retracted article, indicate the article's retracted status in the References list and also include a citation and full reference for the retraction notice.

We checked our previously cited papers and their correspondence reference list, and they are all valid. The following papers are added, to address the comments of the reviewers, in the revised version of the paper.

1. Ding, Y., Kim, M., Kuindersma, S., & Walsh, C. J. (2018). Human-in-the-loop optimization of hip assistance with a soft exosuit during walking. *Science robotics*, *3*(15), eaar5438.
2. Sartori, M., Maculan, M., Pizzolato, C., Reggiani, M., & Farina, D. (2015). Modeling and simulating the neuromuscular mechanisms regulating ankle and knee joint stiffness during human locomotion. *Journal of neurophysiology*, *114*(4), 2509-2527.
3. H. Lee, H. I. Krebs and N. Hogan, "A novel characterization method to study multivariable joint mechanical impedance," 2012 4th IEEE RAS & EMBS International Conference on Biomedical Robotics and Biomechatronics (BioRob), Rome, Italy, 2012, pp. 1524-1529, doi: 10.1109/BioRob.2012.6290301.
4. H. Lee, E. J. Rouse and H. I. Krebs, "Summary of Human Ankle Mechanical Impedance During Walking," in IEEE Journal of Translational Engineering in Health and Medicine, vol. 4, pp. 1-7, 2016, Art no. 2100407, doi: 10.1109/JTEHM.2016.2601613.
5. A. Arami, E. van Asseldonk, H. van der Kooij and E. Burdet, "A Clustering-Based Approach to Identify Joint Impedance During Walking," in IEEE Transactions on Neural Systems and Rehabilitation Engineering, vol. 28, no. 8, pp. 1808-1816, Aug. 2020, doi: 10.1109/TNSRE.2020.3005389.
6. Ramadan, R., Geyer, H., Jeka, J. et al. A neuromuscular model of human locomotion combines spinal reflex circuits with voluntary movements. Sci Rep 12, 8189 (2022). https://doi.org/10.1038/s41598-022-11102-1.
7. Aoi, S., Ohashi, T., Bamba, R. et al. Neuromusculoskeletal model that walks and runs across a speed range with a few motor control parameter changes based on the muscle synergy hypothesis. Sci Rep 9, 369 (2019). https://doi.org/10.1038/s41598-018-37460-3.
8. J. Weng, E. Hashemi and A. Arami, "Human Gait Cost Function Varies With Walking Speed: An Inverse Optimal Control Study," in IEEE Robotics and Automation Letters, vol. 8, no. 8, pp. 4777-4784, Aug. 2023, doi: 10.1109/LRA.2023.3289088.
9. H. Dinovitzer, M. Shushtari and A. Arami, "Feedforward Control of Lower Limb Exoskeletons: Which Torque Profile Should We Use?," in IEEE Robotics and Automation Letters, vol. 9, no. 1, pp. 382-389, Jan. 2024, doi: 10.1109/LRA.2023.3331674.

Reviewer 1

This paper presents further work on the VER method. The VER allows control design in the phase space, where the control input is guided towards the limit cycle. This introduces several advantages, like time independence, coordinated motion, phase synchronization, etc.

Overall, I think the control method is very interesting and should be explored further. I also looked at the papers [38] and [39]. I believe the main flaw of the current work is the missing comparison to a "classical" controller with recorded trajectories. The controller is shown to improve the user effort compared to the passive movement and a lot of advantages are mentioned. But this does not really show that such "coordinated" controller is better than a standard, recorded trajectory one. Why is there no such comparison?

Thanks for this comment. To address this, we ran another experiment including three participants and compared the VER controller with a conventional phase-based path (PBP) controller equipped with a robust gait phase estimator. The experimental results indicate a similar kinematic and control performance for both controllers with a significant average total muscle effort reduction of 13.9%, 15.1% and 7.0% using VER controller for participants one, two and three, respectively, compared to phase-based path controller. The results are added to Section 4.2 (page 12-14) and the added experiment protocol is described in Section 3.1.2 (page 7-8); please check the revised version of the paper.

Or, alternatively, a better explanation on how one can measure the coordination between the exoskeleton and the user would help strengthen the claims.

Thanks for raising this interesting point. To the best of our knowledge, currently, there is no control index to comprehensively evaluate and compare the controller performance in rehabilitation scenarios. In the revised version, we acknowledged that how to compare and measure the performance and user-exoskeleton coordination is still an open question; please check the last sentence of Discussion (page 15). We believe that closeness of assisted gait to those of natural walking in sense of spatiotemporal gait parameters, kinematic and kinetic measures can partially indicate aspects of coordinated assisted gait. In addition, no large increase in muscle activities may be an indicator of human-exoskeleton conflict. We provided more details in the revised version see 4th paragraph of Section 5 (page 14) in the revised version of the paper.

Other major comments:

1 – Introduction: "...still far from optimal due to the unresolved human-exoskeleton coordination problem at the controller level." -> I am not sure how the authors reached this conclusion, since there are a lot of works that currently reach very high "optimal" levels of assistance (metabolic cost reductions) using very straightforward control designs. Although, to my knowledge, these achievements were not yet generalized to different tasks. Or is this statement intended towards the current rehabilitation exoskeletons? In that case, the statement should be written differently.

Thank you for this valid comment. We acknowledge that there has been advances in assistive exoskeletons for power augmentation and for metabolic cost reduction which is important but not the only objective of rehabilitation exoskeletons. However, regarding the assistive exoskeletons

for gait rehabilitation, and assisting motor impaired individuals, the performance of those systems in terms of human-robot coordination, stability, and personalization is far from optimal. According to reviewer's comment, we modified the sentence and added a citation to one of the significant works on human in the loop optimization of exoskeleton torque profile that results in reduction of metabolic cost and another advancement on the effect of different feedforward controllers.

- Ding, Y., et al. (2018). Human-in-the-loop optimization of hip assistance with a soft exosuit during walking. *Science robotics*, *3*(15), eaar5438.
- H. Dinovitzer, et al. (2024), "Feedforward Control of Lower Limb Exoskeletons: Which Torque Profile Should We Use?," in IEEE Robotics and Automation Letters, vol. 9, no. 1, pp. 382-389, doi: 10.1109/LRA.2023.3331674.

2 – Introduction: "It has been shown that passive trajectory tracking controllers worsen human-exoskeleton coordination." – there should be a citation here. And I think this is an important statement, since this paper tries to improve on this coordination problem.

We added the citation [15]; please check the revised version of the Introduction (page 2).

Israel et al. , "Metabolic costs and muscle activity patterns during robotic-assisted and therapist-assisted treadmill walking in individuals with incomplete spinal cord injury," Physical therapy 2006, 86(11), 1466-1478.

3 – Figure 2 – a-d are identical from [39] and should be noted as such in the figure caption.

This point is added in the caption of Figure 2.

4 – Section 2.4 "However, to maximize the similarity of the resultant gait to natural walking, the knee and hip limit cycles are designed similar to natural walking; see Fig.2 a-d." I understood only after looking at the [39] that the desired trajectories/limit cycles are modified to be stable. Is there any indication how this modification affects the gait?

The hip natural and modified trajectory and accordingly limit cycles are almost the same, we just make it smoother during stance phase. But, regarding the knee joint, we modified the knee trajectory and consequently its limit cycle during the stance phase. The knee trajectory correction leads to removing the knee flexion during the stance phase, in turn, the VER controller encourages the participants to keep their knee straight during the stance phase, which is similar to many exoskeleton controllers (e.g., Indego) used in clinical settings. This explanation is also added to the revised version of the paper; please see Section. 2.4 (page 6).

5 - How is the "Passive" mode realized. Is the exoskeleton in a zero torque mode or is it completely turned off? Please add a sentence to clarify this. This is important, assuming that the controller is completely turned off, even having friction compensation, would undoubtedly reduce the muscle activations in the active mode.

The "Passive" mode is a zero torque mode and exoskeleton is on. We clarify this in the revised version; please see Section 3.1.1 (about lines 11-12).

6 – In the experimental results, the protocol is explained again. I think this is not needed, since the experimental protocol is written in section 3.1

Thanks for pointing this out. To prevent any redundancy, this part is removed from the experimental results section.

7 – Section 4.2, Kinematics, second paragraph. Only the mass of the exoskeleton is considered to affect the gait. How about kinematic restriction imposed by the exoskeleton, like the simplified passive ankle, and DoF knee and hip?

The reviewer point is valid. We revised this sentence and described other factors that make the user's pattern deviate from natural gait; please see Section 4.1.2 (page 9) in the revised manuscript.

8 – The idea about the central generator is interesting. But I would argue that more literature review in this area would be required to support this statement.

Thanks for this comment. There are several modeling frameworks used to represent human gait control. This includes the studies on human joint mechanical impedance identification during the gait based on external disturbances, neuromuscular reflex models, muscle synergy models and optimal control framework that uses inverse optimal control techniques to discover the underlying cost function of movement. More clarifications and citations are added to the revised paper; please see the last sentences of Section 4.1.3 (page 11).

These citations are added

- H. Lee, et al. (2012)"A novel characterization method to study multivariable joint mechanical impedance," 2012 4th IEEE RAS & EMBS International Conference on Biomedical Robotics and Biomechatronics (BioRob), Rome, Italy, pp. 1524-1529, doi: 10.1109/BioRob.2012.6290301.
- H. Lee, et al. (2016), "Summary of Human Ankle Mechanical Impedance During Walking," in IEEE Journal of Translational Engineering in Health and Medicine, vol. 4, pp. 1-7 , Art no. 2100407, doi: 10.1109/JTEHM.2016.2601613.
- M. Sartori et al. (2015). Modeling and simulating the
  - neuromuscular mechanisms regulating ankle and knee joint stiffness during human
  - locomotion. Journal of neurophysiology. 114(4):2509–2527.
- A. Arami, et al. (2020), "A Clustering-Based Approach to Identify Joint Impedance During Walking," in IEEE Transactions on Neural Systems and Rehabilitation Engineering, vol. 28, no. 8, pp. 1808-1816, doi: 10.1109/TNSRE.2020.3005389.
- R. Ramadan et al. (2022) A neuromuscular model of human locomotion combines spinal reflex circuits with voluntary movements. Sci Rep 12, 8189. https://doi.org/10.1038/s41598-022-11102-1.
- S. Aoi, et al. (2019) Neuromusculoskeletal model that walks and runs across a speed range with a few motor control parameter changes based on the muscle synergy hypothesis. Sci Rep 9, 369. https://doi.org/10.1038/s41598-018-37460-3.

- J. Weng, et al. (2023) "Human Gait Cost Function Varies With Walking Speed: An Inverse Optimal Control Study," in IEEE Robotics and Automation Letters, vol. 8, no. 8, pp. 4777-4784, Aug. 2023, doi: 10.1109/LRA.2023.3289088.

9 – Section 5, second paragraph. What is meant with the absence of conflicts?

We acknowledge that the term was used in an ambiguous form. To prevent miss-misunderstandings this term is removed now.

10 – Section 5, last paragraph. "To sum up,..." sentence is strangely written, please consider revising it.

The sentence is revised.

Minor comments:

- Section 2.2, line 8, there is a typo "...is almost zero, hence"

Thanks for pointing this out. It is corrected.

Reviewer 3

The authors should add the main conclusions of the study in their Abstract.

Thanks for the reviewer's comment. The main conclusions and experimental results are added in the Abstract of the revised paper.

I recommend adding Figure S11 (Supporting material) to the content of the main text of the paper, as it illustrates the exoskeleton used, helping the understanding of Plos One readers.

This figure is now moved to the main body of the paper (Fig 3 in the revised version).

Figure captions are too long. I suggest keeping only a brief presentation of the content, leaving more detailed descriptions for the main text of the paper.

We have done our best summarizing most of the figure captions. Please see Fig. 1, 4, 6,
