# Supplementary material for: Coordinated human-exoskeleton locomotion emerges from regulating virtual energy
Source: PLoS One. 2025 Jan 22;20(1):e0292334. doi: 10.1371/journal.pone.0292334 (PMC11753675; doi:10.1371/journal.pone.0292334)
Supplement: Attachment — Submitted filename: Rebuttal Letter (2).pdf [file pone.0292334.s006.pdf]

The authors would like to thank the Associate Editor and Reviewers for their valuable comments on our manuscript, Submission ID PONE-D-23-27913R1, entitled "Coordinated human-exoskeleton locomotion emerges from regulating virtual energy". In the following, you may find our responses to the questions and comments of the reviewers. We addressed all the reviewers' comments one by one in this response letter and revised the manuscript accordingly. Changes are highlighted in the attached copy of the revised manuscript at the end of this document to facilitate finding the relevant modifications to each comment. The highlighted captions also show the revised captions and/or figures.

Best Regards,

Rezvan Nasiri, Hannah Dinovitzer, Nirosh Manohara, and Arash Arami

University of Waterloo

Journal Requirements

Please review your reference list to ensure that it is complete and correct. If you have cited papers that have been retracted, please include the rationale for doing so in the manuscript text, or remove these references and replace them with relevant current references. Any changes to the reference list should be mentioned in the rebuttal letter that accompanies your revised manuscript. If you need to cite a retracted article, indicate the article's retracted status in the References list and also include a citation and full reference for the retraction notice.

The following citations were added due to the reviewers' request.

- Chen, B., Ma, H., Qin, L. Y., Gao, F., Chan, K. M., Law, S. W., ... & Liao, W. H. (2016). Recent developments and challenges of lower extremity exoskeletons. *Journal of Orthopaedic Translation*, *5*, 26-37.
- Lee, H., Ferguson, P. W., & Rosen, J. (2020). Lower limb exoskeleton systems—overview. *Wearable Robotics*, 207-229.

Additional Editor Comments

Please carefully examine the revision of the Reviewer 2 and provide point-to-point answers to resolve the remaining issues. Take in consideration also the typos addressed by the Reviewer 1.

We addressed all the reviewers' points carefully, and revised the paper accordingly.

Reviewer 1

1. I noticed that there are many typos in the paper. I would suggest the authors read the paper again and correct them. Here are some that I found:
   "by" and "has" in line "Given gait variability, which can be intensified by an impairment, intention decoding based on gait kinematics has not been successful so far [33-35] despite the recent achievements in real-time gait phase estimation [36-39]."
   "Straighten" in line "Accordingly, we removed the flexion behavior of the knee joint during the stance phase; hence, the controller encourages the participants to straighten their leg during the stance."

"Experiment" in "The testing sessions were different for each experiment."
"were" in "Nine participants (7 male and 2 female, age: 23.9 ± 3.2 years, body mass: 72.3 ± 7.0 kg, height: 177 ± 6.3 cm; mean±standard deviation) participated in the Experiment 1."
We like to thank the reviewer for pointing out those issues; we now corrected all the typos. In addition, we proofread the paper carefully to rectify any English issues.

Reviewer 2

The paper discusses an interesting case of using VER. The authors have earlier worked on the topic and have extended it here with human experimentation. The manuscript is written well and present a thought out study. However, there are a few points that need more work.

2. The authors provide a reference to their earlier works in the introduction to establish the contribution of the current paper. It appears that the concept has been developed earlier and a feasibility one human study has been published. Thus, the main contribution of the current work is implementation of the same over multiple subjects (n=9). This makes the current contribution of the paper weaker as only 9 subjects were tested, and no disabled walking studies were conducted.
   Thanks for this comment. Please note that, our previous work included a theoretical and simulation work (referring to RA-L paper) and a conference paper with only one participant with a different focus which was the ability of VER in estimating gait phase (a relatively short conference paper). That one participant experiment was only a proof of concept and did neither follow similar experimental protocol or data collection and analysis, for instance it did not include any muscle activity (EMG) data.
   The main contributions of the presented paper are: (1) experimenting the controller for the first time on a group of 9 able-bodied individuals, (2) vast experimental data analysis in terms of kinematics, controller command, questionnaire, and muscle activity of the users and comparing the VER resultant gait with natural walking, (3) comparing the controller with an effective controller presented in the filed in terms of kinematic analysis and muscle EMG reduction, (4) presenting a new metric to measure the individuals contribution to the gait as well as presenting the analytical convergence proof by considering the user active contribution to the gait, and hypnotizing that human lower limb neuromuscular system works similar to the VER controller. We believe that the provided points make the contributions of this paper solid and distinct. Moreover, we clearly mentioned in the discussion (now changed its name to Conclusion as the discussions are merged to the Results and Discussion section) that the presented controller is not applied on motor-impaired individuals, and it is considered as our future work. According to reviewer's comment, we revised the Abstract to clarify the contributions of this work; please check the Abstract of the revised Conclusion.

3. Also, it is not clear how the experiments 1 and 2 design strengthen the point that such approach can be useful with disabled walking.
   Thanks for this comment. Our controller is not aiming to help individuals who cannot contribute to the motion at all, but those with residual ability to move (for instance,

individuals with incomplete spinal cord injury or stroke). The main challenge for those individuals with various motor impairments is active contribution to motion to regain their ability to walk again and the main drawback of the existing controllers is the point that the lower limb exoskeleton controllers mainly cannot coordinate with human gait variability. Accordingly, this paper focused on analyzing the gait naturalness and coordination performance of the resultant gait by VER controller. Experiment 1 is designed to study the VER performance in a group of 9 healthy individuals. In this experiment, the treadmill speed is selected similar to the walking speed suggested for individuals with motor impairments [22-30] to test it closer to the needs of those individuals. Having said that we acknowledge that this work is a step needed to build the foundation for next studies on people with those motor disabilities. This is now further clarified in the revised Conclusion section.

Experiment 2 is also designed to compare our controller with the path controller [22-24], which is a well-known and effective controller in the rehabilitation field and was experimented on a wide range of individuals with motor impairment [22,23], in terms of joint kinematics and muscle EMG reduction. Also note that this experiment was added to this work as suggested by another reviewer in the previous round. In the revised version of the paper, we added a new paragraph at the beginning of the Experiment Design section to explain our perspectives in design of experiment 1 and experiment 2.

4. The second major comment is that the discussion section is very weak. Considering that the introduction section introduces the readers to the various strategies being tested in the community working on human in the loop, adaptive controllers, etc. the discussion section does not establish the proposed uniqueness and advantages as claimed for this work. This needs to be addressed.
   Thanks for this comment. We noticed that some parts of our discussion were already within the Results section. As removing those parts and adding them to the separate Discussion section could undermine the readability of this paper, we decided to join the Discussion and Results sections as Results and Discussion section.
   The revised Results and Discussion section includes the analysis and discussion of human-robot interaction with a focus on muscle activity analysis, and the analysis of kinematics, gait spatiotemporal parameters, perceived quality of interaction through questionnaire, and the analysis of naturalness of assisted gait. We also discussed the control performance of VER, and compared it to path controller equipped with a robust gait phase estimator.
   While our Introduction mentioned other controllers in the field to set the stage for the VER in this domain, the comparison of VER with various control strategies is beyond the scope of this work. This is now clarified in the Conclusion section. However, as also asked by another reviewer in the previous round we have already ran new experiments and added the results and comparison with path controller tested on three individuals, as one of the successful controllers in the gait rehabilitation field (as a reminder Experiment 2 was added later to the paper to enrich the results and conclusion).
   The Conclusion section, while mostly focused on summarizing the main experimental results and achievements of the paper, is now revised according to the reviewer’s comment to clarify the limitations and the future work.

5. A claim "that passive trajectory tracking controllers worsen human-exoskeleton coordination" is made using reference [15] - this is an old reference. Are there newer references supporting this claim?
   The following paper is cited as [16] to address the reviewer's concern.
   - Chen, B., Ma, H., Qin, L. Y., Gao, F., Chan, K. M., Law, S. W., ... & Liao, W. H. (2016). Recent developments and challenges of lower extremity exoskeletons. *Journal of Orthopaedic Translation*, *5*, 26-37.

6. Section 2.4 puts "Lack of actuation at the ankle joint in our exoskeleton results in walking pattern slightly different than natural walking. ... natural walking, the knee and hip limit cycles are designed similar to natural walking ..." This needs to be discussed further as it is not clear how this impacts the performance and how this will be taken care of disabled walking.
   Most of the existing lower limb assistive exoskeletons are using the passive ankle joints, since having an active ankle joint makes the exoskeleton bulky (impractical for those users with residual ability to move) and expensive [22], please also see the review paper below, which is now cited as [44]. In addition, in controller failure cases, active ankles may lead to a fall or major injury for the individuals with motor impairment. Accordingly, considering the cost efficiency, applicability, and safety limitations, our lower limb assistive exoskeleton also utilizes the passive ankle joints. Hence, only hip and knee joints are left to be controlled.
   In most of the existing controllers for lower limb assistive exoskeletons, the knee and hip reference motions are designed similar to human normal walking and may be slightly modified to address the exoskeleton and controller limitations. Accordingly, the trajectories are similar but not identical to able-bodied individual's motion profiles; please check the following review paper.
   The same scenario was also considered in the design of the reference trajectory leading to the limit cycles for hip and knee in our controller. We clarified these points in the revised version of Mathematics section; please check section 2.4 in the revised paper. The mentioned review paper was also added to the paper.
   - Lee, H., Ferguson, P. W., & Rosen, J. (2020). Lower limb exoskeleton systems—overview. *Wearable Robotics*, 207-229.
